# LR12 Promotes Liver Repair by Improving the Resolution of Inflammation and Liver Regeneration in Mice with Thioacetamide- (TAA-) Induced Acute Liver Failure

**DOI:** 10.1155/2021/2327721

**Published:** 2021-05-28

**Authors:** Yongjuan Wang, Xiaoli Xie, Hongqun Liu, Huimin Liu, Huiqing Jiang

**Affiliations:** ^1^Department of Gastroenterology, The Second Hospital of Hebei Medical University, Hebei Key Laboratory of Gastroenterology, Hebei Institute of Gastroenterology, Hebei Clinical Research Center for Digestive Diseases, China; ^2^Liver Unit, Cumming School of Medicine, University of Calgary, Calgary, Canada

## Abstract

**Background:**

Triggering receptor expressed on myeloid cells-1 (TREM-1) controls the mobilization of inflammatory cells in response to injury and consequently enhances liver damage. LR12 is a TREM-1 inhibitory peptide. However, the role of LR12 in acute liver failure (ALF) has remained elusive. This study was aimed at indicating whether LR12 could promote liver repair in mice with thioacetamide- (TAA-) induced ALF.

**Methods:**

BALB/c mice were intraperitoneally injected with TAA, followed by intravenous injection of LR12. Damage and regeneration of the liver were assessed. LO2 cells and macrophages were used to assess the therapeutic effects of LR12.

**Results:**

Mice treated with TAA for 24 h developed ALF, while liver inflammation was alleviated after LR12 treatment. Moreover, LR12 promoted hepatocyte regeneration in mice with TAA-induced ALF. *In vitro*, the supernatant from TAA+LR12-treated macrophages promoted the proliferation of LO2 cells. Cytokine protein microarray analysis suggested that LR12 promoted the secretion of C-C chemokine ligand 20 (CCL20) from macrophages. Besides, neutralization of CCL20 blocked the effects of LR12, thus inhibited the proliferation of LO2 cells *in vitro*, aggregated the liver inflammation, and restrained hepatocyte regeneration in ALF mice *in vivo*. Furthermore, we also found that LR12 activated the p38 mitogen-activated protein kinase (MAPK) pathway in hepatocytes through promoting the secretion of CCL20 from macrophages.

**Conclusions:**

LR12 could improve the resolution of inflammation and liver regeneration in mice with TAA-induced ALF by promoting the secretion of CCL20 from macrophages and activating the p38 MAPK pathway. Therefore, LR12 could be an attractive therapeutic target for the treatment of ALF.

## 1. Introduction

Acute liver failure (ALF) is a highly unpredictable disease that can evolve into a fatal consequence within few days or weeks. Although it can be induced by a variety of factors [1–6], it is characterized by similar clinical features, including acute deterioration of liver function, extensive necrosis of hepatocytes, and multiple organ failure. To our knowledge, ALF seriously threatens human health because of the high mortality rate and the lack of an effective medication [7, 8]. Liver transplantation is the most effective treatment for ALF [9, 10]. However, its application has been restricted by a serious shortage of donor organs and high expenses. Therefore, novel effective therapies are urgently required.

Hepatic necrosis leads to the recruitment of a large number of inflammatory cells in the liver, which further triggers the systemic inflammatory response syndrome, and may be the main pathological mechanism of ALF [11, 12]. Therefore, inhibition of inflammatory response is essential for the treatment of ALF. Triggering receptor expressed on myeloid cell-1 (TREM-1) is mainly expressed on macrophages, neutrophils, and monocytes and may act as a cell surface receptor, transcribing proinflammatory cytokines and interacting with toll-like receptors (TLRs) [13, 14]. Recently, studies have shown that TREM-1 promotes liver inflammation, and deletion of TREM-1 reduces liver injury and inflammatory cell infiltration [15, 16]. Small molecules and peptides, such as LP17 and LR12, can inhibit TREM-1. As a conserved motif from a TREM-like transcript, LR12 (LQEEDAGEYGCM) reduces the inflammatory response caused by lipopolysaccharide (LPS), prevents connective tissue disorders, and even improves the survival rate [17]. Additionally, LR12 inhibits the amplification loop of the inflammatory process mediated by TREM-1, while it does not eliminate the inflammatory response [18]. The safety and pharmacokinetics of LR12 (http://www.inotrem.com; MOTREM™, INN: nangibotide, CAS number 2014384-91-7) are being evaluated in a first-in-man study. However, its role in the treatment of ALF has remained elusive.

Previous studies have demonstrated that liver regeneration is a critical determinant of survival in animal models of ALF [19, 20]. Agents enhancing liver regeneration hold a great therapeutic potential for ALF. A study has shown that blockade of TREM-1 potentiates cellular proliferation in an experimental model of ischemic stroke, resulting in functional improvement [21]. The present study was aimed at indicating whether LR12 could alleviate liver inflammation and promote liver regeneration in thioacetamide- (TAA-) induced ALF in mice.

## 2. Materials and Methods

### 2.1. Animal Models

Male Balb/c mice (age, 6-8 weeks old) were attained from Beijing Vital River Laboratory Animal Technology Co., Ltd. (Beijing, China) and fed with food and water ad libitum under 12 : 12 h light-dark cycles. The random assigning of mice into four groups was undertaken as follows: control, TAA model, LR12, and TAA+LR12 (*n* = 6/group). PBS was infused into mice in the control group; TAA (1200 mg/kg, Beijing Solarbio Science & Technology Co., Ltd., Beijing, China) was intraperitoneally (i.p.) injected in the TAA model group; LR12 (5 mg/kg, Beijing SBS Genetech Co., Ltd., Beijing, China) was intravenously administrated in the LR12 group; and TAA (1200 mg/kg) was i.p. injected, followed by intravenous injection of LR12 (5 mg/kg) 1 h later in the TAA+LR12 group. The mice utilized for survival analysis were randomly allocated into two groups: TAA model and TAA+LR12 (*n* = 9/group). The mice used for administration of the anti-C-C chemokine ligand 20 (CCL20) antibody were randomly assigned into two groups: TAA+LR12+rat IgG (1 mg/kg/day, i.p.; R&D Systems, Minneapolis, MN, USA) and TAA+LR12+anti-CCL20 (1 mg/kg/day, i.p.; R&D Systems) (*n* = 4/group).

All animal experiments were carried out according to the requirements of the Ethics Committee of the Second Hospital of Hebei Medical University (Shijiazhuang, China; approval no. 2021-AE004).

### 2.2. Cell Lines and Cultivation

The human hepatic LO2 cells and human monocytic THP-1 cells were achieved from Shanghai Institutes for Biological Sciences of Chinese Academy of Sciences (Shanghai, China). Cultivation of LO2 and THP-1 cells into a Roswell Park Memorial Institute- (RPMI-) 1640 medium was conducted with the addition of 10% fetal bovine serum (FBS) (Gibco, Carlsbad, CA, USA). Differentiation of THP-1 monocytes into macrophages was undertaken after stimulating with 100 nM phorbol 12-myristate 13-acetate (PMA) for 72 h. Then, the cells were cultured in the RPMI-1640 medium for 72 h. LO2 cells were assigned into four groups: control, 0.5 mM, 1 mM, and 2 mM group. LO2 or THP-1 cells were divided at random into four groups: control, TAA model, LR12, and TAA+LR12. The control group received PBS; the TAA model group was stimulated with TAA (1 mM); the LR12 group received LR12 (10 ng/mL); and the TAA+LR12 group was administrated with TAA (1 mM) and LR12 (10 ng/mL). LO2 cells for CCL20 (100 ng/mL) treatment were allocated into four groups: control, TAA model, CCL20, and TAA+CCL20. LO2 cells for anti-CCL20 treatment were allocated at random into two groups: TAA+LR12 and TAA+LR12+anti-CCL20 (5 mM). A humidified atmosphere (5% CO_2_, 37°C) was utilized for the cultivation of cell lines.

### 2.3. Isolation and Culture of Primary Hepatocytes and Macrophages

It was attempted to perfuse the liver with ethylenediaminetetraacetic acid (EDTA, 37°C, 5 min), as well as digest it with the assistance of 0.1% pronase E (37°C, 2 min) and 0.032% collagenase IV (37°C, 5 min). After the collagenase perfusion, the digested liver was removed and manually disrupted with ophthalmic scissors in a DMEM containing 10% FBS and 1% penicillin-streptomycin. The tissue was then filtered through a 70 *μ*m nylon mesh. It was attempted to undertake centrifugation (150 g, 4°C, 10 min). The pellet was enriched with hepatocytes. The supernatant (enriched with macrophages) was transferred into a clean EP tube, centrifugation was performed (150 g, 4°C, 10 min), the pellet was suspended with 3 mL DMEM, and 30% percoll (4 mL) was added into the DMEM. After centrifugation (500 g, 5 min), the middle layer enriched with macrophages was collected, mixed with 4 mL DMEM, and centrifuged. The pellet enriched with hepatocytes was suspended with DMEM (6 mL), 50% percoll (5 mL) was added into the DMEM, and centrifugation was followed (150 g, 5 min). After that, the pellet (enriched with hepatocytes) was suspended with William's E medium and cultured at the same environment as macrophages. Primary hepatocytes and macrophages were divided at random into four groups: control, TAA model, LR12, and TAA+LR12.

### 2.4. Blood Chemistry and Liver Histology

The levels of alanine aminotransferase (ALT) and aspartate aminotransferase (AST) in serum were detected using kits that could be attained from the Nanjing Jincheng Bioengineering Institute (Nanjing, China). We employed hematoxylin and eosin (H&E) to stain slides (5 *μ*m), and the visualization was undertaken via a light microscope (Olympus, Japan).

### 2.5. Confocal Laser Scanning Microscopy (CLSM)

CLSM was carried out as per standard protocol. Primary antibodies were as follows: anti-cytokeratin 18 (CK-18) (Proteintech Group, Chicago, IL, USA), antiproliferating cell nuclear antigen (PCNA) (Proteintech Group), anti-F4/80 (Santa Cruz Biotechnology, Dallas, TX, USA), and anti-CCL20 (Affinity Biosciences, Cincinnati, OH, USA). We also utilized the ImageJ software (National Institutes of Health, Bethesda, MD, USA) to perform analyses.

### 2.6. Western Blotting

We further attempted to homogenize liver samples and cells in radioimmunoprecipitation assay (RIPA) buffer. Western blotting was undertaken as per standard protocol. Primary antibodies were as follows: PCNA (1 : 1000), p-p38 (1 : 500; Abways Co., Ltd., Shanghai, China), p38 (1 : 500; Abways), glyceraldehyde 3-phosphate dehydrogenase (GAPDH) (1 : 1000; Abcam, Cambridge, UK), and beta-actin (1 : 1000). Visualization of protein bands was undertaken via the ECL system and imaged using the Odyssey Infrared Imaging System (LI-COR, Lincoln, NB, USA), which was standardized to beta-actin or GAPDH as internal controls.

### 2.7. Cell Counting Kit-8 (CCK-8) Assay

Evaluation of cell survival rates was carried out using the CCK-8 assay (KeyGen Biotechnology Co., Ltd., Nanjing, Jiangsu, China). Seeding of ~10^4^ cells into 96-well plates was conducted. After 24 h of culture, different doses of TAA and LR12 (10 ng/ml) or supernatant from macrophages treated with TAA+LR12 were added separately. Incubation of each well was undertaken with the assistance of 10 *μ*g CCK-8 in the dark (2 h). We also could measure absorbance (490 nm) via a multifunction microplate reader (BioTek, Montpellier, VT, USA).

### 2.8. Flow Cytometry (FCM)

Seeding of 1 × 10^6^ LO2 cells into 6-well plates was conducted. Supernatants from macrophages treated with TAA or TAA+LR12 were added into LO2 cells. After 24 h of culture, LO2 cells were digested with trypsin and centrifuged at 1000 × *g* for 5 min. Cells were washed with PBS and fixed with 70% ethanol at 4°C overnight. Cells were then centrifuged at 1000 × *g* for 5 min and rewashed with PBS. After gentle mixing with PI (Beyotime, Shanghai, China), mixed cells were filtered and incubated in the dark at 37°C for 30 min before analysis with a flow cytometer (BD Biosciences, Franklin Lake, New Jersey, USA). PI fluorescence was detected at 488 nm.

### 2.9. Cytokine Protein Microarray

The supernatants from the four groups (control, TAA model, LR12, and TAA+LR12) were collected. The RayBio Human Cytokine Antibody Array Kit (QAH-TH17-1-1 microarray; RayBiotech, Norcross, GA, USA) was utilized to identify the cytokines based on instructions provided by the manufacturer. The images were analyzed via the RayBio ScanAnalyzer software (RayBiotech).

### 2.10. Enzyme-Linked Immunosorbent Assay (ELISA)

The concentration of CCL20 was determined by using the 70-EK1211-24 kit (MultiSciences Co., Ltd., Hangzhou, China) based on instructions provided by the manufacturer. We also measured the optical density through an enzyme-labelling reader (450 nm) with a 570 nm wavelength correction reading. The concentrations of the unknown reagents were measured as well.

### 2.11. Quantitative Reverse Transcription Polymerase Chain Reaction (RT-qPCR)

The isolation of total RNA from cells was undertaken with the RNeasy Mini Kit (Qiagen, Hilden, Germany). The synthesis of first-strand cDNA from 2 *μ*g RNA was carried out via the iScript cDNA Synthesis Kit (Bio-Rad Laboratories, Inc., Hercules, CA, USA). RT-qPCR was applied in triplicate using the SYBR Green Real-Time PCR Master Mix System. Normalization of gene expression data was performed with the assistance of GAPDH. The following sequences of primers were used for RT-qPCR: for hCCL20: 5′-3′ “TTGCTCCTGGCTGCTTTGATGT” and 3′-5′ “GTTTTGGATTTGCGCACACAGAC,” and for hGAPDH: 5′-3′ “CAACGGATTTGGTCGTATTGG” and 3′-5′ “GCAACAATATCCACTTTACCAGAGTTAA.” The changes of gene expression data were calculated by the 2^−ΔΔCT^ method.

### 2.12. Statistical Analysis

We employed the SPSS 25.0 software (IBM, Armonk, NY, USA) to conduct statistical analyses. We also attempted to present data in the form of mean ± standard deviation (SD). We utilized one-way analysis of variance (ANOVA) for comparing data among multiple groups. We set the significance level to *P* < 0.05.

## 3. Results

### 3.1. LR12 Attenuated Liver Injury and Promoted Liver Regeneration in Mice with TAA-Mediated ALF

**Figure 1:**
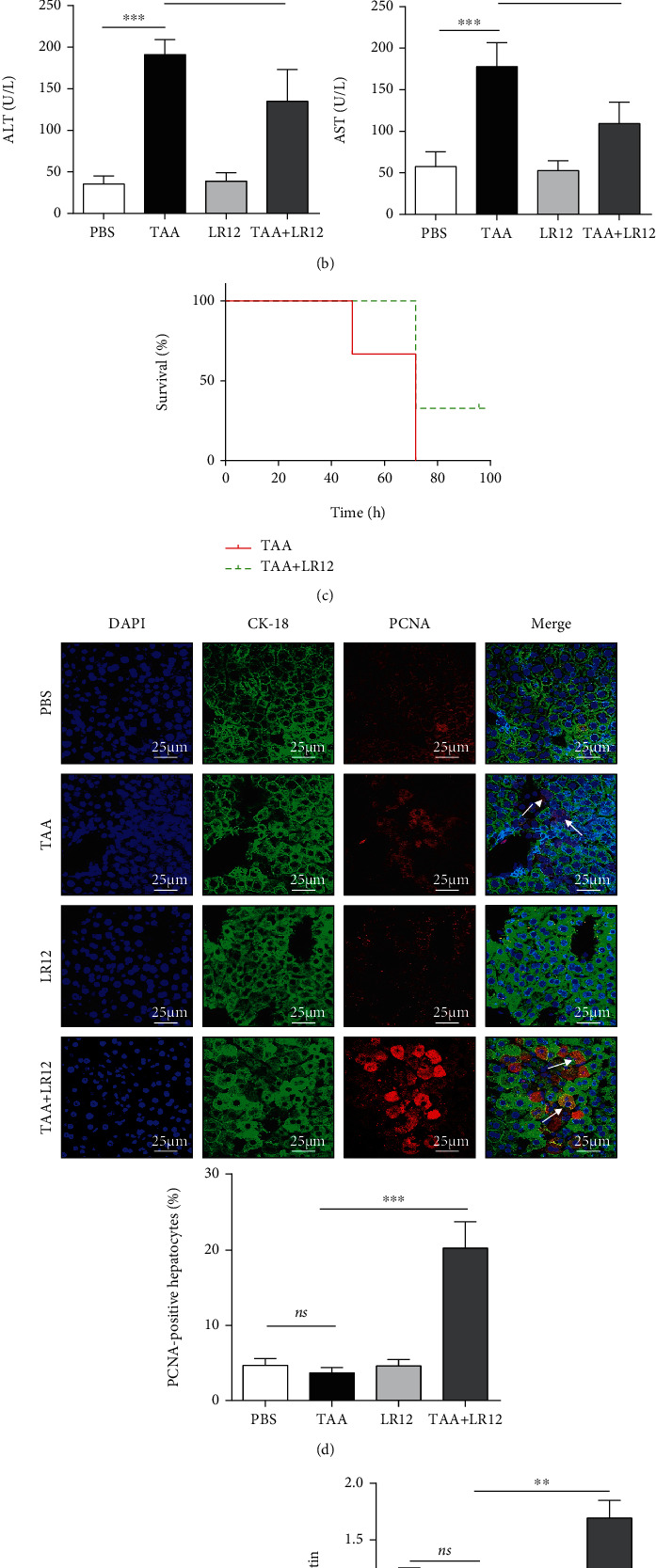
Injection of LR12 reduced the liver inflammation and stimulated liver regeneration following TAA-induced ALF in mice. (a) H&E staining (magnification, 200x and 400x) showing inflammatory cell infiltration and necrotic area (%) in the liver. (b) Serum levels of alanine aminotransferase (ALT) and aspartate aminotransferase (AST). (c) Survival curve from mice with TAA-induced ALF (*n* = 9/group). (d) CLSM showing CK18 and PCNA staining in the liver. (e) Western blotting detected PCNA expression in the liver tissue. Data were presented as the mean ± standard deviation (SD) (*n* = 6). ^∗^*P* < 0.05, ^∗∗^*P* < 0.01, and ^∗∗∗^*P* < 0.001 versus control group.

There were no significant inflammatory cell infiltration and hepatic necrosis in the liver in control and LR12 groups. Compared with the control group, hepatic necrosis was evident in the liver in mice with TAA-induced ALF, accompanied by inflammatory cell infiltration (Figure 1(a)). After LR12 treatment for 24 h, the necrosis and inflammation in the liver were significantly reduced (Figure 1(a)). Additionally, the serum levels of ALT and AST in the TAA model group were markedly elevated compared to those in the control group. However, they were significantly downregulated in the TAA+LR12 group (Figure 1(b)). Most importantly, we tested the mortality of mice at time points of 48, 72, and 96 h, and the findings revealed that LR12 prolonged the survival time of mice compared with the TAA model group (Figure 1(c)). CLSM indicated that compared to the TAA model group, PCNA-positive hepatocytes in the liver in the TAA+LR12 group were significantly upregulated, which were mainly located in a central venous catheter (Figure 1(d)), and this result was in line with the outcome of Western blotting (Figure 1(e)).

### 3.2. LR12 Did Not Promote Proliferation by Directly Acting on Hepatocytes

**Figure 2:**
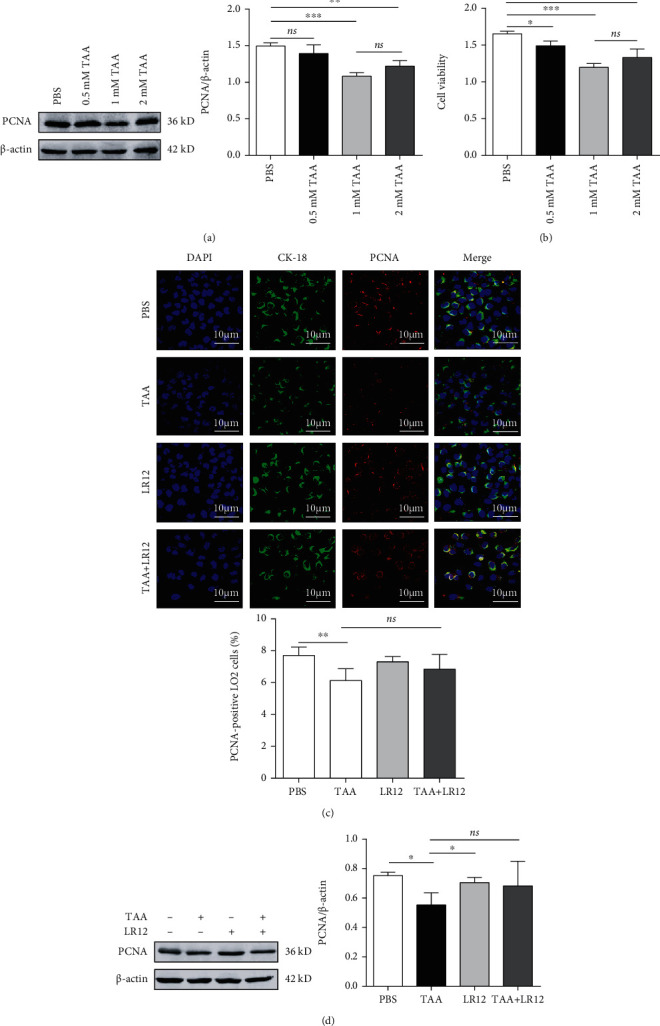
TAA induced hepatic injury *in vitro* and LR12 did not directly promote the proliferation of hepatocytes. (a) Western blot analysis of PCNA in LO2 cells stimulated with different concentrations of TAA (0.5, 1, and 2 mM). (b) CCK-8 assay of viability of LO2 cells. (c) CLSM showing CK18 and PCNA staining in LO2 cells. (d) Western blot analysis of PCNA expression in LO2 cells stimulated with TAA (1 mM) and/or LR12 (10 ng/mL). Data were presented as the mean ± standard deviation (SD). ^∗^*P* < 0.05, ^∗∗^*P* < 0.01, and ^∗∗∗^*P* < 0.001 versus control group.

Firstly, we established a model of hepatic injury in LO2 cells *in vitro* that could be treated with diverse concentrations of TAA (0, 0.5, 1, and 2 mM), and the outcomes of Western blotting uncovered that PCNA expression was downregulated in both 1 mM and 2 mM groups (Figure 2(a)). Besides, the CCK-8 assay demonstrated that the proliferation of LO2 cells was notably attenuated in the three mentioned concentrations (Figure 2(b)). Although there was a mild increase in both PCNA and CCK-8 viability of LO2 cells in the 2 mM group, no significant difference was found in comparison with that in the 1 mM group (Figures 2(a) and 2(b)). In order to indicate whether LR12 could directly promote the proliferation of hepatocytes, we used 1 mM TAA to induce hepatic injury and then treated LO2 cells with LR12 (10 ng/mL). The results of Western blotting and CLSM unveiled that PCNA expression was not elevated in LO2 cells in the TAA+LR12 group (Figures 2(c) and 2(d)), which confirmed that LR12 did not promote proliferation by directly acting on hepatocytes.

### 3.3. LR12 Promoted the Proliferation of Hepatocytes by Acting on Macrophages

**Figure 3:**
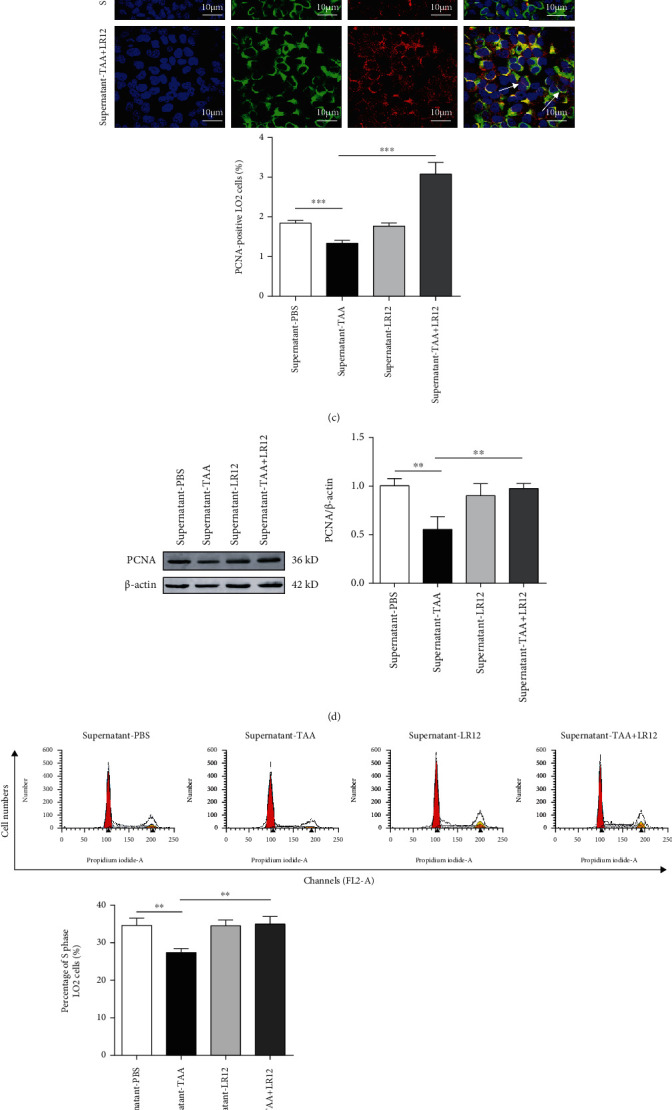
LR12 promoted hepatocyte proliferation via macrophages in LO2 cells. (a) THP-1 cells were successfully differentiated into macrophages by PMA (100 ng/mL). (b) Immunofluorescence images of F4/80 staining and DAPI in macrophages. (c) CLSM showing CK18 and PCNA staining in LO2 cells stimulated with the macrophage supernatant. (d) Western blot analysis of PCNA in LO2 cells stimulated with the macrophage supernatant. (e) The LO2 cell cycle detected by flow cytometric analysis. (f) CCK-8 assay analysis of LO2 cell viability. Data were presented as the mean ± standard deviation (SD). ^∗^*P* < 0.05, ^∗∗^*P* < 0.01, and ^∗∗∗^*P* < 0.001 versus control group.

**Figure 4:**
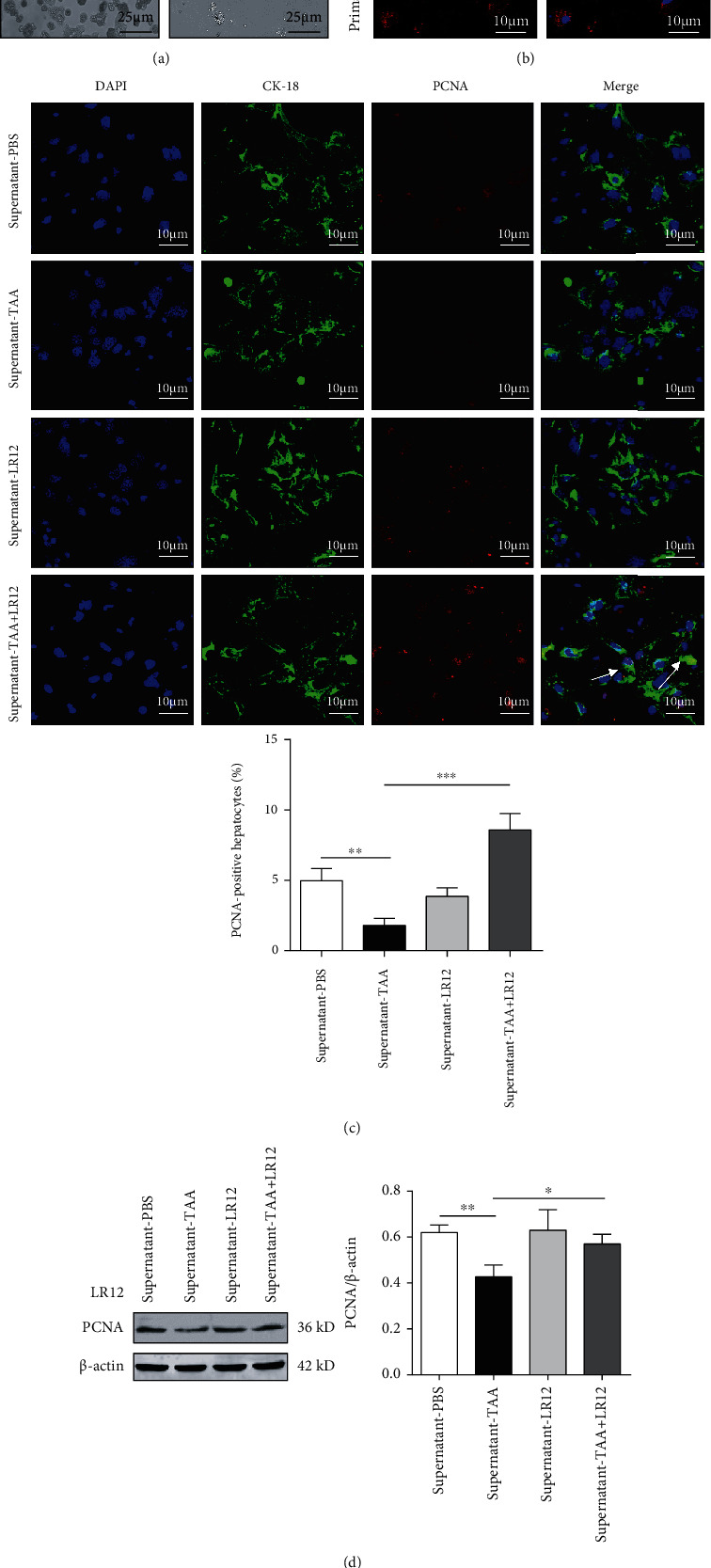
LR12 promoted primary hepatocyte proliferation via macrophages. (a) Primary hepatocytes and macrophages were extracted. (b) Immunofluorescence images of F4/80 staining and DAPI in primary macrophages. (c) CLSM showing CK18 and PCNA staining in primary hepatocytes stimulated with the primary macrophage supernatant. (d) Western blot analysis of PCNA in primary hepatocytes stimulated with the primary macrophage supernatant. Data were presented as the mean ± standard deviation (SD). ^∗^*P* < 0.05, ^∗∗^*P* < 0.01, and ^∗∗∗^*P* < 0.001 versus control group.

To better understand the mechanisms of LR12 promoting the proliferation of hepatocytes, we attempted to determine whether it could act on macrophages. Firstly, we differentiated THP-1 cells into macrophage-like cells. Our results showed that PMA (100 ng/mL) successfully induced the adhesion of THP-1 cells (Figure 3(a)). In addition, immunofluorescence staining revealed that the adherent THP-1 cells were F4/80-positive cells (Figure 3(b)). We treated LO2 cells with TAA (1 mM) for 24 h and then treated macrophages with TAA (1 mM) combined with LR12 (10 ng/mL, 24 h), and we subsequently transferred the supernatant of macrophages into LO2 cells (24 h). CLSM and Western blotting uncovered that PCNA expression in LO2 cells treated with the supernatant from TAA-treated macrophages was downregulated; besides, the supernatant from TAA+LR12-treated macrophages significantly upregulated PCNA expression in LO2 cells (Figures 3(c) and 3(d)). In addition, FCM demonstrated that the supernatant from TAA+LR12-treated macrophages induced a higher number of LO2 cells in the S phase (Figure 3(e)). The CCK-8 assay indicated that the proliferation of LO2 cells was significantly elevated by the supernatant from TAA+LR12-treated macrophages (Figure 3(f)). The testing of primary hepatocytes and macrophages revealed similar results as LO2 cells and THP-1-induced macrophages (Figure 4).

### 3.4. LR12 Induced Regeneration of Hepatocytes by Promoting the Secretion of CCL20

**Figure 5:**
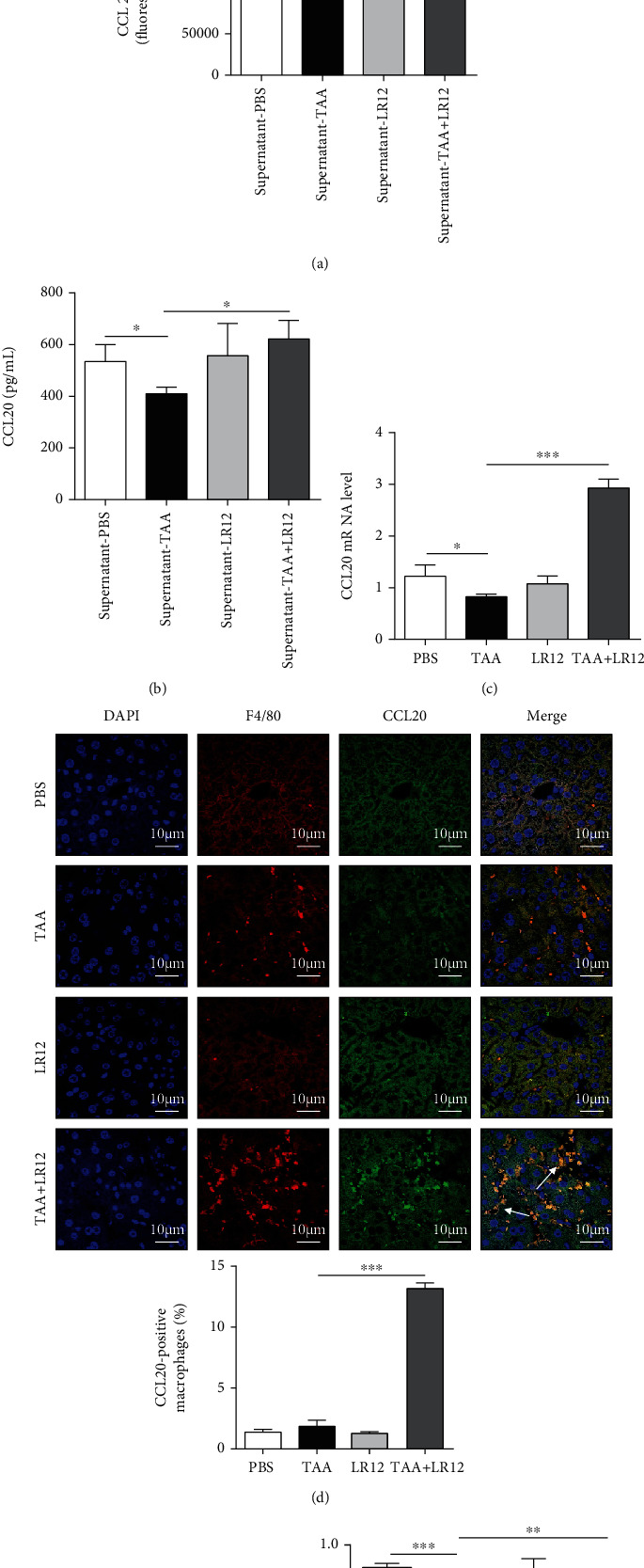
LR12 promoted hepatocyte regeneration via CCL20 secreted by macrophages. (a) The cytokine protein microarray of the supernatant from macrophages. (b) ELISA of CCL20 in the supernatant from macrophages. (c) The mRNA level of CCL20 in macrophages. (d) CLSM showing F4/80 and CCL20 staining in the liver tissues. (e) Western blot analysis of PCNA in LO2 cells stimulated with CCL20. Data were presented as the mean ± standard deviation (SD). ^∗^*P* < 0.05, ^∗∗^*P* < 0.01, and ^∗∗∗^*P* < 0.001 versus control group.

The results of cytokine protein microarray revealed that the protein level of CCL20 in the supernatant from TAA-treated macrophages was downregulated (Figure 5(a)). However, the protein level of CCL20 was significantly upregulated in the supernatant from TAA+LR12-treated macrophages (Figure 5(a)). ELISA also revealed that the protein level of CCL20 was downregulated in the supernatant from TAA-treated macrophages, while it was upregulated in the supernatant from TAA+LR12-treated macrophages (Figure 5(b)). We also found that the CCL20 mRNA level was markedly downregulated in TAA-treated macrophages compared to the control group, while it was upregulated in TAA+LR12-treated macrophages (Figure 5(c)). In addition, CLSM indicated that compared to the TAA model group, CCL20 expression in F4/80-positive cells was upregulated in mice treated with TAA+LR12 (Figure 5(d)). Furthermore, CCL20 (100 ng/mL) upregulated the PCNA expression in TAA-treated LO2 cells *in vitro* (Figure 5(e)).

### 3.5. CCL20 Neutralization Aggravated the Severity of TAA-Induced ALF and Inhibited Liver Regeneration

**Figure 6:**
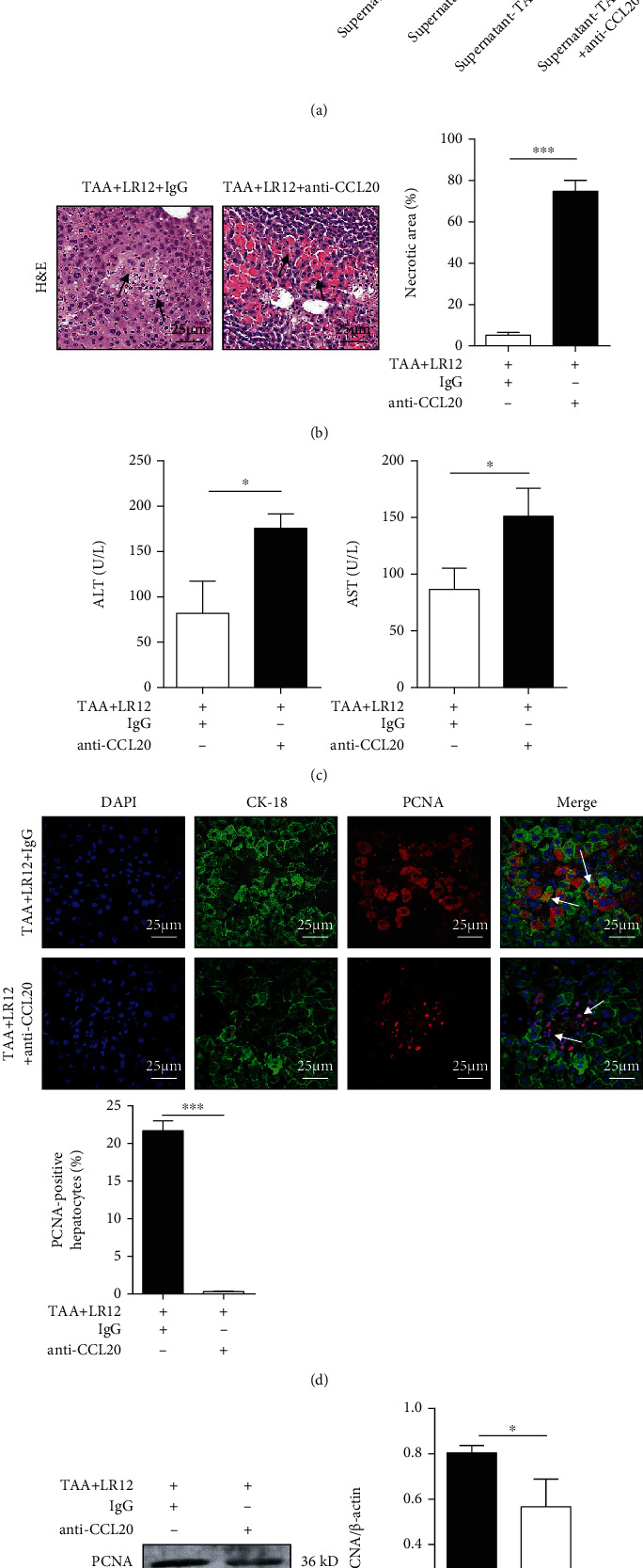
CCL20 neutralization aggravated the severity of TAA-induced ALF and inhibited liver regeneration. (a) Western blot analysis of PCNA in LO2 cells stimulated with TAA+LR12 or/and anti-CCL20 hAbs. (b) H&E staining (magnification, 400x) of the liver tissues in mice treated with TAA+LR12 and normal rat IgG or anti-CCL20 mAbs. (c) The levels of alanine aminotransferase (ALT) and aspartate aminotransferase (AST) in serum. (d) CLSM showing CK18 and PCNA staining in the liver tissues in mice treated with TAA+LR12 and normal rat IgG or anti-CCL20 mAbs. (e) Western blot analysis of PCNA in the liver tissues. Data were presented as the mean ± standard deviation (SD). ^∗^*P* < 0.05, ^∗∗^*P* < 0.01, and ^∗∗∗^*P* < 0.001 versus control group.

Western blotting indicated that compared to TAA+LR12-treated LO2 cells, anti-CCL20 hAbs (5 mM) blocked the effects of LR12 on macrophages and downregulated PCNA expression in LO2 cells *in vitro* (Figure 6(a)). Anti-CCL20 mAbs (1 mg/kg) also significantly aggravated the severity of hepatic necrosis and increased the serum levels of ALT and AST in the TAA+LR12 group (Figures 6(b) and 6(c)). In addition, CLSM showed that neutralization of CCL20 inhibited PCNA expression in hepatocytes, while it upregulated PCNA expression in inflammatory cells (Figure 6(d)). This finding was also approved by Western blotting (Figure 6(e)).

### 3.6. LR12 Activated the p38 Mitogen-Activated Protein Kinase (MAPK) Signaling Pathway by Promoting CCL20 Secretion from Macrophages

**Figure 7:**
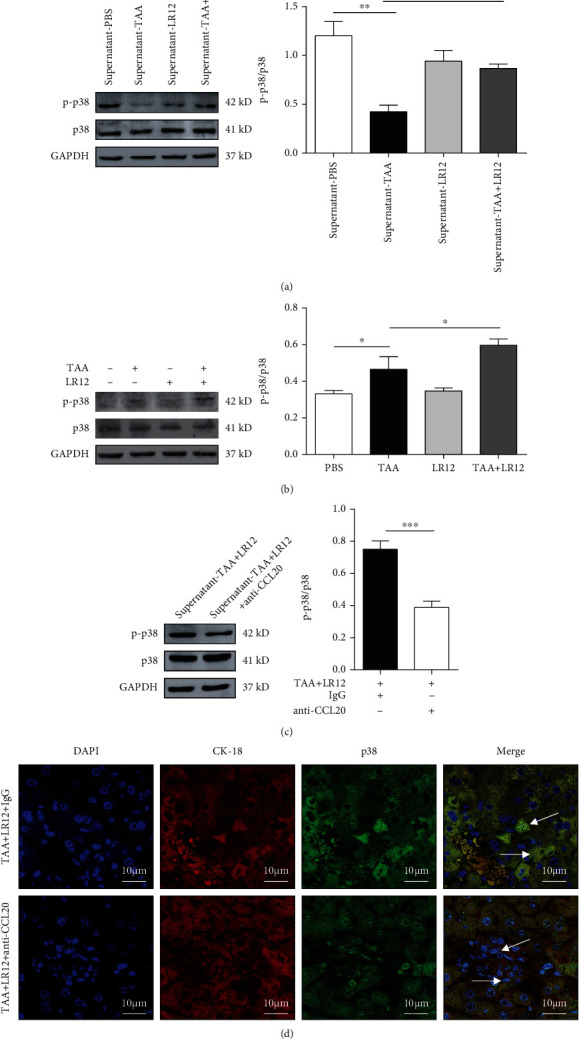
LR12 activated the p38 MAPK pathway by promoting the secretion of CCL20 from macrophages. (a) Western blot analysis of p-p38 in LO2 cells stimulated with the supernatant from macrophages. (b) Western blot analysis of p-p38 in primary hepatocytes. (c) Western blot analysis of p-p38 in LO2 cells stimulated with TAA+LR12 or/and anti-CCL20 hAbs. (d) CLSM showing CK18 and p38 staining in the liver tissues. Data were presented as the mean ± standard deviation (SD). ^∗^*P* < 0.05, ^∗∗^*P* < 0.01, and ^∗∗∗^*P* < 0.001 versus control group.

The phosphorylation level of p38 (p-p38) MAPK expression was downregulated in the TAA group, and LR12 treatment led to a noticeable increase in p-p38 MAPK (Figure 7(a)). We further extracted primary hepatocytes after treatment with LR12 in mice with TAA-induced ALF. Our findings unveiled that p-p38 MAPK level was markedly elevated in the TAA+LR12 treatment group (Figure 7(b)). In addition, anti-CCL20 hAbs downregulated the protein level of p-p38 MAPK in the TAA+LR12 group *in vitro* (Figure 7(c)). CLSM showed that LR12 enhanced translocation of p38 MAPK from the cytoplasm to the hepatocyte nuclei in mice, while anti-CCL20 mAbs inhibited the accumulation of p38 MAPK in hepatocyte nuclei and upregulated the protein level of p38 MAPK in the inflammatory cells in LR12-treated ALF mice (Figure 7(d)).

## 4. Discussion

**Figure 8:**
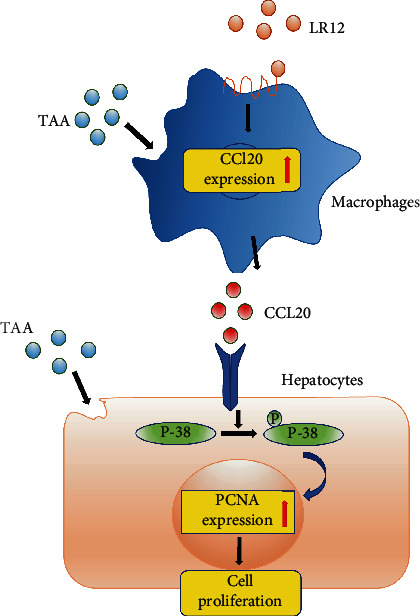
LR12 could promote regeneration of hepatocytes.

At present, this is the first study concentrated on the role of LR12 in the promotion of liver regeneration on TAA-induced ALF in mice via macrophages. The anti-CCL20 antibody blocked the effects of LR12 on hepatocyte proliferation. In addition, the present research indicated that LR12 activated the p38 MAPK pathway in hepatocytes via CCL20 secreted by macrophages (Figure 8).

TREM-1 is known to amplify inflammatory responses. Recently, evidences showed that it played an important role in sterile inflammatory diseases. Nguyen-Lefebvre et al. reported that TREM-1 promoted liver injury induced by carbon tetrachloride (CCl_4_) administration [15]. Rao et al. demonstrated that inhibition of TREM-1 attenuated lipid accumulation and inflammation in the nonalcoholic fatty liver disease [16]. Another research showed that TREM-1 inhibitors could prevent excessive inflammation and decrease the severity of the diseases and mortality. LR12 could reduce the severity of sepsis and acute myocardial infarction [22]. Consistent with previous studies, the present study revealed that LR12 reduced the liver inflammation and hepatic necrosis in mice with TAA-induced ALF. Most importantly, LR12 could improve the survival rate of ALF mice.

To date, a limited number of researches have been concentrated on the relationship between TREM-1 and tissue regeneration. Xu et al. showed that blockade of TREM-1 potentiated synaptic plasticity and cellular proliferation in the hippocampus in an experimental model of ischemic stroke, which resulted in a long-term functional improvement [21]. In the current research, we found that LR12 could elevate PCNA expression and thus could promote regeneration of damaged hepatocytes in mice with TAA-induced ALF. To our knowledge, regenerative nodules are commonly observed in chronic liver injury, such as cirrhosis, which can be formed by local proliferation of hepatocytes and deposition of the extracellular matrix [23]. In our study, we found that the regenerated hepatocytes were located in the area of hepatic necrosis, and the structure of hepatic lobules in the TAA+LR12 group was normal, without deposition of the extracellular matrix in the central vein, which was different from the regenerative nodules in the cirrhotic liver.

In order to better study the mechanism of LR12 promoting hepatocyte regeneration, we established a cell model of TAA-induced hepatic injury *in vitro*, and we found that TAA could inhibit the PCNA expression and cell viability in LO2 cells. However, our results showed that LR12 could not directly promote the regeneration of hepatocytes in TAA-induced hepatic injury *in vitro*. The liver contains an abundant population of tissue-resident macrophages, namely, Kupffer cells (KCs), accounting for 80-90% of the body's macrophages [24]. Due to their broad diversity and plasticity, KCs play dual roles in different stages of ALF [25]. In the early phase of ALF, hepatocyte-mediated release of alarmins triggers the activation of KCs to produce a large amount of proinflammatory cytokines, thereby aggravating tissue damage [24]. However, in the resolution/tissue repair phase of ALF, KCs undergo functional reprogramming toward a prorestorative phenotype, favoring wound healing and tissue recovery [26–28]. Lewis and Veldhuizen found that macrophages may hold a therapeutic value for acetaminophen- (APAP-) induced ALF to facilitate resolution of necrosis and promote liver regeneration [29]. Golbar et al. showed that the depletion of hepatic KCs by chlorophosphonic acid liposomes could significantly aggravate TAA-induced ALF in rats [30]. In the current study, we treated LO2 cells with the supernatant from macrophages stimulated with TAA or TAA+LR12, and we found that LR12 could increase the expression of PCNA in LO2 cells, which indicated that LR12 promoted liver regeneration through macrophages in TAA-induced hepatic injury *in vitro*. Additionally, our results indicated that the expression of PCNA was the highest in the TAA+LR12 group in mice with TAA-induced ALF, while a significantly higher expression of PCNA was not observed in the TAA+LR12 group *in vitro*. This may be related to the fact that hepatocytes are in a state of temporary nonproliferation *in vivo*; thus, the expression of PCNA was not significantly increased in the control or LR12 group in mice. However, LO2 cells are immortalized cell lines, with a strong proliferation ability under normal conditions.

Macrophages and macrophage-related factors have been reported to play a substantial role in the liver regeneration. A previous research demonstrated that perfluorooctane sulfonate could induce the activation of KCs in hepatocyte proliferation through the nuclear factor kappa B (NF-*κ*B)/tumor necrosis factor-alpha (TNF-*α*)/IL-6 signaling pathway [31]. The results of the current research unveiled that the levels of cytokines (e.g., TNF-*α*, IL-6, and transforming growth factor-*β* (TGF-*β*)) did not increase significantly. However, we found that CCL20 expression was upregulated after LR12 treatment. CCL20, as a second-generation chemokine, may act as both homeostatic and inflammatory chemokines under different conditions. It was originally identified in the liver [32]. The role of CCL20 in injury and regeneration of the liver remains controversial. Affo et al. reported that CCL20 played an important role in inducing the direct damage to the liver cells and/or participates in the LPS cascade, leading to the liver injury [33]. However, many scholars pointed out that the liver-homing capacity of CCL20 may also mediate tissue protection, repair, and regeneration. Rao et al. demonstrated that *γδ*T cells regulated liver regeneration by producing IL-22 and IL-17, which could not only accelerate the direct mitosis of hepatocytes but also promote the regeneration of leukocytes in the liver [34]. Scheiermann et al. reported that utilization of the IL-36 receptor antagonist weakened the CCL20 expression and impaired liver regeneration in APAP-induced liver injury in mice [35]. Our findings demonstrated that CCL20 upregulated PCNA expression in TAA-induced hepatic injury in Lo-2 cells *in vitro*, which indicated that CCL20 might promote liver regeneration in mice with TAA-induced ALF. Recently, Gonzalez-Guerrero et al. showed that neutralization of CCL20 in acute kidney injury might aggravate kidney injury and was associated with a reduced cell proliferation [36]. In the present study, we found that neutralization of CCL20 blocked the effects of LR12 on macrophages and inhibited the proliferation of hepatocytes *in vitro*. We also noted that neutralization of CCL20 increased the liver inflammation and liver necrosis in the TAA+LR12 group. Importantly, we observed that neutralization of CCL20 inhibited the effects of LR12 and downregulated the expression of PCNA in hepatocytes in mice with TAA-induced ALF. Hence, it could be concluded that LR12 promoted hepatocyte regeneration by stimulating macrophages to secrete CCL20.

To date, the role of the p38 MAPK pathway in the injury and regeneration of the liver has remained elusive. Heinrichsdorff et al. reported that the p38 MAPK pathway inhibited the activation of JNK, thereby preventing liver failure that might be caused by LPS or TNF-*α* [37]. Zhang et al. declared that IL-18 promoted the proliferation of liver cells in rat through the p38 MAPK signaling pathway [38]. However, Campbell et al. concluded that the p-p38 MAPK pathway was not necessary for DNA replication during liver regeneration after partial hepatectomy [39]. Tormos et al. demonstrated that p38 played a dual role in the proliferation of hepatocytes [40]. The present research uncovered that LR12 induced p-p38 MAPK expression in hepatocytes in TAA-induced ALF. However, neutralization of CCL20 inhibited p-p38 MAPK expression in LO2 cells. Therefore, our findings suggested that LR12 promoted liver regeneration via CCL20, as well as activating the p38 MAPK pathway.

In summary, we first addressed the role of LR12 in TAA-induced ALF in mice. Furthermore, LR12 could promote liver regeneration, mainly by promoting macrophages to secrete CCL20 and activating the p38 MAPK pathway. Therefore, our results indicated that LR12 could be an attractive therapeutic target for the treatment of ALF.

## Data Availability

The data in this study is available upon reasonable request to the correspondence author.

## Abbreviations

ALF: Acute liver failure
ALT: Alanine aminotransferase
AST: Aspartate aminotransferase
APAP: Acetaminophen
CCL20: C-C chemokine ligand 20
CLSM: Confocal laser scanning microscopy
CCl_4_: Carbon tetrachloride
CK18: Cytokeratin 18
CCK-8: Cell Counting Kit-8.
ELISA: Enzyme-linked immunosorbent assay
EDTA: Ethylenediaminetetraacetic acid
FCM: Flow cytometry
GAPDH: Glyceraldehyde 3-phosphate dehydrogenase
H&E: Hematoxylin and eosin
KCs: Kupffer cells
LPS: Lipopolysaccharide
MAPK: Mitogen-activated protein kinase
NF-*κ*B: Nuclear factor kappa B
PMA: Phorbol 12-myristate 13-acetate
PCNA: Proliferating cell nuclear antigen
RIPA: Radioimmunoprecipitation assay
RT-qPCR: Quantitative reverse transcription polymerase chain reaction
SD: Standard deviation
TAA: Thioacetamide
TREM-1: Triggering receptor expressed on the myeloid cell-1
TLRs: Toll-like receptors
TNF-*α*: Tumor necrosis factor-alpha
TGF-*β*: Transforming growth factor-*β*.
